# The psychometrics of the Persian version of the perfectionistic self‐presentation scale–junior form

**DOI:** 10.1002/brb3.3074

**Published:** 2023-05-16

**Authors:** Faezeh Peimanpak, Abbas Abdollahi, Simin Hosseinian

**Affiliations:** ^1^ Department of Counseling, Faculty of Education and Psychology Alzahra University Tehran Iran

**Keywords:** adolescents, children, perfectionism, perfectionistic self‐presentation, psychopathology, reliability, validity

## Abstract

**Introduction:**

The perfectionistic self‐presentation scale (PSPS)–junior form is a self‐report questionnaire used to measure perfectionistic self‐presentation in children and adolescents. It consists of 18 items and 3 subscales, including perfectionistic self‐promotion, non‐display of imperfection, and nondisclosure of imperfection.

**Methods:**

The present study aimed to determine the psychometrics of the Persian version of the PSPS. A descriptive study was conducted on 345 samples (269 girls) who responded to the questionnaire.

**Results:**

The findings confirmed the internal consistency and composite reliability (CR) of this scale (CR = 0.744). Further, the Persian PSPS has acceptable face and content validities. Construct and convergent validities were also measured and confirmed by confirmatory factor analysis. The correlational analysis of the research variables showed that the PSPS is positively correlated with the Child‐Adolescent Perfectionism Scale (0.566) and the children's and adolescents’ dysfunctional attitudes scale (0.420).

**Conclusion:**

Overall, the results indicated that the Persian version of the PSPS has acceptable psychometrics and can yield accurate results in Iranian samples.

## Graphical abstract

The present study aimed to determine the psychometrics of the Persian version of the PSPS. The findings confirmed the internal consistency and composite reliability of this scale the results indicated that the Persian version of the PSPS has acceptable psychometrics and can yield accurate results in Iranian samples.

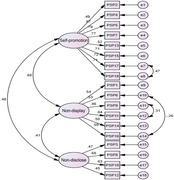

## 1 INTRODUCTION

Perfectionism is a multifaceted characteristic associated with efforts to be perfect, setting very high standards for one's performance, and an overly critical self‐evaluation of one's behaviors (Egan et al., 2022). Hewitt et al. (2008) considered perfectionism to be a neurotic/maladaptive personality type consisting of three major personal and interpersonal components; the first component is the trait dimensions of perfectionism, which pertain to one's need to be perfect for oneself or others. Such characteristics are known as self‐oriented perfectionism (obligating oneself to be perfect), other‐oriented perfectionism (obligating others to be perfect), and socially prescribed perfectionism (perceiving that others obligate us to be perfect). The second component of this model is the interpersonal expression of perfectionism or feeling encouraged to appear flawless to others through openly promoting perfection or hiding one's imperfection. This component is also referred to as perfectionistic self‐presentation and has three sub‐components, including perfectionistic self‐promotion (actively promoting one's assumed perfection), non‐display of imperfection (avoiding the potential display of imperfection to others/one's associates), and nondisclosure of imperfection (avoiding the verbal disclosure of imperfection to others). Finally, the third component encompasses cognitive processes that reflect information processing based on perfectionistic cognitive structures and automatic perfectionistic thoughts. All these components are associated with different psychopathologies, accomplishments, and relationship problems (Hewitt et al., 2008).

According to the perfectionism social disconnection model (Hewitt et al., 2017), perfectionism develops as a result of a lack of fit or asynchronous parent–child relationship where the child's needs for security, nurturance, and support have been either inadequately or inconsistently met or completely neglected. As such, the PSDM proposes that the child engages in perfectionistic behaviors (i.e., needing to be or appear perfect) to secure acceptance and to repair a sense of defectiveness or “inner badness,” as being perfect and/or appearing to be perfect is believed to gain approval and acceptance from people around them. From this perspective, early attachment insecurities and feelings of defectiveness/shame and the negative self‐models they represent are at the core of perfectionism and perfectionistic behaviors.

The present study focuses on perfectionistic self‐presentation based on the new criteria developed for children and adolescents. Perfectionistic self‐presentation is a concept stemming from the observations that some perfectionists need to outwardly appear perfect, whereas, in fact, these individuals often view themselves to be far from perfection. Therefore, they hide behind a mask of excellence and perfection (Hewitt et al., 2011). Hewitt et al. (2003) introduced perfectionistic self‐presentation as a form of interpersonal expression. The construct of perfectionistic self‐presentation is related to one's interpersonal goals and desires. That is, the way in which people interact with others typically represents an attempt to portray the self favorably (Baumeister & Leary, 1995). Furthermore, perfectionistic self‐presentation is a form of self‐presentation, in which the desire to present that self as favorably becomes maladaptive.

Recently, Hewitt et al. (2011) created a version of this measure that is suitable for use with children and adolescents. The development of this scale accords with descriptions of some children being high in public self‐consciousness and displaying a “false front” to compensate for vulnerable self‐esteem. Hewitt et al. (2011), to initial research conducted with multiple samples on the development of a multidimensional measure of perfectionistic self‐presentation for children and adolescents, developed an 18‐item version of the perfectionistic self‐presentation scale (PSPS). Analyses conducted on data from two clinical samples and one nonclinical sample of children and adolescents found that the PSPS–junior form (PSPS–JR) reflected a multidimensional model of perfectionistic self‐presentation with three subscales: perfectionistic self‐promotion, non‐display of imperfection, and nondisclosure of imperfection. The subscale scores were found to demonstrate internal consistency, and there was good evidence supporting the validity of the interpretation of subscale scores based on this new measure. The psychometric properties of the scale were further assessed by the authors in a follow‐up study with a younger sample of children in grades seven and eight (Fellet et al., 2012). The study assessed the psychometric characteristics and correlates of the PSPS–JR. The findings support the continued use of the PSPS–JR and the assessment of individual differences in perfectionistic self‐presentation among early adolescents.

In summary, there are too few studies to be able to recommend this measure for a younger sample. Confirmation of its factor structure and examination of its test–retest reliability is required by independent researchers. The PSPS–JR was designed for use with children and adolescents, but its psychometric properties and applications among Iranian adolescents have not been investigated. Research with the PSPS–JR is still in its early phases, and several psychometric and substantive issues remain to be examined.

Perfectionism studies have expanded considerably over the past two decades, investigating several factors concerning this component, particularly in terms of its essence, correlations, and consequences in children and adolescents. Tendency to distress and incompetence are, to an extent, due to perfectionistic adolescents’ willingness to strive for absolute perfection instead of self‐critical evaluation based on specific standards (Flett et al., 2012). For instance, Bruch (1974) noted that his adolescent patients felt the need to portray perfection and often described the difference between the perfect self‐image they show others, as well as their inner experience of themselves. Other researchers pointed out the self‐image or the mask that is portrayed by particular adolescents who massively invest in creating and maintaining an ideal public self‐image (Bruch, 1974). Peterson (2002) described a concept known as the “façade of invulnerability,” which is a common phenomenon among intellectually capable, yet troubled, youth (Peterson, 2002). He noted that “It is not easy for them to reveal doubts, embarrassments, shame, and feelings of awkwardness,” and therefore, they display a false and idealistic sense of self (Knopf, 2021). Likely, adolescents who portray a false sense of self that is designated to seem flawless are exceptionally self‐aware given their great concerns about social acceptance, social integration, and the avoidance of public failures that are specific to adolescence (Flett et al., 2012).

Growing evidence suggests that perfectionism in children and adolescents is accompanied by numerous adjustment issues and problems, such as depression and anxiety (Wright et al., 2021), obsessive–compulsive disorder (Miegel et al., 2020), bulimic tendencies (Bento et al., 2020), fear/sadness (Flett et al., 2011), suicidal ideation (Pia et al., 2020), and eating disorder symptoms (Vacca et al., 2021). According to the literature, maladaptive forms of perfectionism are prevalent in approximately 30% of adolescents and are demonstrated by 3 out of every 10 adolescents (Vatterott, 2019).

Huang et al. (2020) believed that perfectionistic presentation leads to negative thoughts, threatening the mental health of adolescents (Huang et al., 2020). Moreover, Lin et al. (2019) demonstrated that adolescents who pretend to be perfect frequently experience sleep disorders due to negative attitudes (Lin et al., 2019). Friedman (2006) also reported that a considerable number of adolescents have extremely covert behaviors to hide their psychological problems (e.g., suicide attempts) from their parents (Friedman, 2006). Similarly, Sorotzkin (1998) observed that astute children and adolescents are often good at displaying behaviors that are similar to perfectionistic self‐presentation to divert others’ attention to their accomplishments, thereby avoiding feelings of inadequacy and self‐destructive tendencies (Sorotzkin, 1998). Hewitt et al. (2011) stated that perfectionistic self‐presentation corresponds to the accounts given by adolescents who have a generally high level of self‐awareness and are often inclined toward “pretension” as compensation for their vulnerable self‐esteem. Along with hiding one's inadequacies, this tendency may lead to severe consequences in anxious adolescents. Perfectionistic self‐presentation is, in fact, a contributing factor to suicide without an evident warning (Hewitt et al., 2011).

The adverse consequences of perfectionism in the lives of youth (children and adolescents) are now widely recognized, including impact on mental health and general well‐being. In order to develop interventions to prevent and treat perfectionism and promote mental health for children, rigorous testing and examination of theoretical models are needed as well as having access to valid and reliable assessment tools. With this background and given the lack of a scale to measure perfectionistic self‐presentation in the Iranian population, the present study aimed to evaluate the psychometrics of the PSPS–JR in a sample of Iranian adolescents. Further, the convergent validity (CV) of the questionnaire was compared to the Child‐Adolescent Perfectionism Scale (CAPS) and the children's and adolescents’ dysfunctional attitudes scale (DAS‐CA). Assessing the psychometrics of the PSPS in other populations can expand the use of this scale.

## 2 METHOD

### 2.1 Participants

This was a descriptive–correlational study. The sample population included adolescent boys and girls, and 345 subjects were selected to complete the questionnaire. There were 269 girls (78%) aged 15–18 years (mean: 16.25 ± 0.77 years) among the samples. In terms of discipline, 113 subjects (32.56%) studied literature and humanities, 86 (24.78%) studied natural sciences, 80 (23.05%) studied mathematics and physics, and 68 subjects (19.59%) studied vocational courses/arts. The inclusion criteria to respond to the questionnaire were having basic literacy and consent to participate in the research (self‐report).

### 2.2 Measure

#### 2.2.1 Perfectionistic self‐presentation scale–junior form (PSPS–JR)

It is an 18‐item self‐report scale used to assess perfectionistic self‐presentation in children and adolescents. The items are scored based on a five‐point Likert scale ranging from *Not at All* (1) to *Very Much* (5). Original evidence suggests that the three subscales of perfectionistic self‐promotion (e.g., *I'd like to appear perfect to others*), non‐display of imperfection (e.g., *my mistakes get worse when others notice them*), and nondisclosure of imperfection (e.g., *I must always hide my problems*) have acceptable internal consistency and validity. In the study by Hewitt et al. (2011), Cronbach's alpha coefficients of these subscales were reported to be .93, .81, and .76, respectively (Hewitt et al., 2011).

#### 2.2.2 Child‐Adolescent Perfectionism Scale (CAPS)

It is a 22‐item questionnaire, with 11 items focusing on self‐oriented perfectionism (e.g., *I try to be perfect in everything I do*) and 11 items focusing on socially prescribed perfectionism (e.g., *others expect me to be perfect*). The items of the CAPS are scored based on a five‐point Likert scale ranging from *Completely Disagree* (1) to *Completely Agree* (5) (Flett et al., 2000). The CAPS has been employed in several studies, such as those conducted by Flett et al. (2000) and Hewitt et al. (2002) (Badri et al., 2015; Hewitt et al., 2002). Cronbach's alpha coefficient of .84 indicates the high reliability of the scale (Flett et al., 2000). In an Iranian study, Badri et al. (2014) reported Cronbach's alpha coefficients for self‐oriented perfectionism and socially prescribed perfectionism as .67 and .74, respectively. In the present study, Cronbach's alpha coefficient of the CAPS was estimated at .80.

#### 2.2.3 Children's and adolescents’ dysfunctional attitudes scale (DAS‐CA)

The DAS‐CA was developed by D'Alessandro and Burton (2006). Using the adult version of the dysfunctional attitudes scale (Weissman, 1979; Weissman & Beck, 1978) and simplifying the statements, the researchers first created the 40‐item version of the questionnaire, and the initial analysis of the 40‐item version led to the elimination of 18 items (D'Alessandro & Burton, 2006). To complete the DAS‐CA, the participants respond to the items based on a five‐point Likert scale ranging from *Completely Disagree* (1) to *Completely Agree* (5). Notably, the findings of D'Alessandro and Burton (2006) experimentally confirm the technical features of the DAS‐CA. In another study, Oral and Günlü (2020) reported Cronbach's alpha reliability coefficient to be .82, and the validity coefficient was estimated at .72 based on factor analysis (Tuncay & Günlü, 2020). In an Iranian study, exploratory factor analysis, reliability analyses, and confirmatory factor analysis were undertaken to assess the psychometric properties and validation of the DAS. Exploratory factor analyses showed a four‐factor model of dysfunctional attitude scale. The fit of the proposed four‐factor model was not promising. The internal consistency of the DAS (40 items) was reasonable (Cronbach's α = .72) (Talepasand et al., 2010). Cronbach's alpha coefficient of the scale was .70 in the present study.

### 2.3 Procedure of study

As participation in this study was unpaid, obtaining informed consent from the participants was mandatory. All the stages of the research were conducted under appropriate instructions and regulations. We used the PSPS simultaneous with the CAPS and DAS‐CA, which were previously translated into Persian, and their reliability and validity were confirmed in different studies. The Brislin method was used to translate the PSPS into Persian (Lonner & Berry, 1986). First, two English–Persian translators, who were native Persians, translated the PSPS. Next, the PSPS was translated from English to Persian by two translators. The two translated versions were discussed in a meeting to eliminate contradictions. Following that, the corrected translation was edited by an expert in the Persian language and literature. The edited version was then delivered to two translators who were fluent in the English language to ensure accuracy. Afterward, an English language expert back‐translated the questionnaire into English. Finally, the original, translated, and back‐translated versions of the questionnaire were delivered to a third translator who was fluent in both English and Persian to approve the Persian version of the PSPS. After the final version was approved and ambiguities were resolved, a pilot study was conducted on 20 adolescents who did not participate in the research project to solve any problems in understanding the content of the questionnaire.

Data collection was performed during November–December 2022 after the research subject was approved by the Research Ethics Committee of Alzahra University (IR/11/26/1401). All the participants provided informed consent before responding to a questionnaire that was prepared for completion within 10 min online via the Porsline platform. This demographic questionnaire collected data on the age, gender, and discipline of the participants. Data analysis and the evaluation of the psychometrics of the PSPS were carried out using SPSS version 26 and AMOS version 23.

## 3 RESULTS

### 3.1 Face validity

The face validity of the PSPS–JR was evaluated qualitatively. For this purpose, a panel of five elites and professors of the faculty of psychology were asked to assess the difficulty level, disparity, and ambiguity of the statements/wording. Minor modifications were made to the tool based on the provided feedback. Cohen's Kappa statistic is frequently used to test interrater reliability. The importance of rater reliability lies in the fact that it represents the extent to which the data collected in the study are correct representations of the variables measured. Measurement of the extent to which data collectors (raters) assign the same score to the same variable is called interrater reliability. Cohen's Kappa always ranges between 0 and 1, with 0 indicating no agreement between the two raters and 1 indicating perfect agreement between the two raters. In the present study, the agreement obtained 80%.

### 3.2 Content validity

Content validity determines whether the items of a questionnaire represent all aspects of a construct. It can be measured either qualitatively or quantitatively. For the qualitative assessment of content validity, we asked five professors to suggest corrections (written) after carefully reading the PSPS items. In their qualitative assessment, they were also asked to consider grammatical accuracy, proper diction, the significance of the questions, the proper arrangement of the questions, and the time needed to respond to the designed tool in their qualitative assessment. After obtaining feedback from the experts, necessary changes were made to the scale.

The next stage involved the quantitative assessment of content validity. Content validity ratio (CVR) was used to ensure the significance and correctness of the selected content, and content validity index (CVI) was used to confirm that the questionnaire items were designed optimally for measuring the content. To measure CVR, 12 experts were asked to score each item based on a 3‐point Likert scale, including *Unnecessary* (1), *Useful but unnecessary* (2), and *Necessary* (3). Based on the Lawshe table and considering the number of experts on the panel, if the index is larger than 62% for an item, the item is necessary and significant at *p* < .05 (Lawshe, 1975). According to the findings, the coefficients of all the PSPS items were within the range of 0.733–1 and equal to 0.837 for the entire scale, confirming its acceptable content validity.

CVI is measured based on three criteria, namely, simplicity, relevance, and clarity, based on a four‐point Likert scale, including irrelevant (1), need serious review (2), relevant but need review (3), and completely related (4). The score of each item should be above 0.79 (Waltz & Bausell, 1981). In the present study, the CVI of all the items was within the range of 0.866–1, and it was equal to 0.947 for the entire PSPS, both of which are acceptable.

### 3.3 Data analysis

As the data were gathered online, there were no outliers. Data normality was assessed based on skewness and kurtosis. According to the findings, the skewness of the questionnaire items ranged from −1.002 to −0.031, and kurtosis ranged from −1.14 to 0.688. Considering the acceptable range of skewness and kurtosis (±2) (George, 2011), data normality was confirmed. In the continuation of the evaluation, the values of kurtosis and skewness related to the Mahalanobis distance and checking the box plot diagram and removing outliers finally showed that the distribution of multivariate data was normal.

### 3.4 Construct validity

To examine the construct validity of the PSPS, we used maximum likelihood confirmatory factor analysis in AMOS. As the factor load of items should be higher than 0.4 (Kline, 2015), the questionnaire items were tested to eliminate those with a lower factor load from further analysis. According to the results, the factor load of the items was within the range of 0.445–0.794, and they were all considered significant (*p* < .01). Therefore, none of the items were eliminated (see Table 1).

**TABLE 1:** Descriptive statistic and factor loadings

Items	*M*	SD	Factor loadings
1. I always have to look as good as I can	4.04	.959	**0.493**
4. It is important to act perfectly around other people	3.50	1.13	**0.672**
5. I always have to look perfect	3.24	1.15	**0.794**
7. I have to look perfect when I am around others	3.37	1.14	**0.771**
13. If I seem perfect, other people will like me more	3.09	1.18	**0.524**
15. I have to look like I always do things perfectly	3.46	1.06	**0.711**
17. I try hard to look perfect around other people	3.58	1.11	**0.724**
18. I like trying to look perfect to other people	3.52	1.10	**0.710**
**Self‐promotion**	27.80	6.45	**–**
1. I think a lot about mistakes that I have made in front of other people	3.70	1.09	**0.542**
6. I feel bad about myself when I make mistakes in front of other people	3.56	1.11	**0.531**
9. I want others to know about it when I do something well	3.13	1.18	**0.462**
11. Mistakes are worse when others see me make them	3.37	1.07	**0.643**
14. I do not want my friends to see even one of my bad points	3.32	1.25	**0.503**
16. It would be bad if I made a fool of myself in front of other people	3.58	1.12	**0.591**
**Non‐display**	20.92	4.38	**–**
3. I do not let other people know when I fail at something	3.49	1.10	**0.468**
8. I should always keep my problems secret	2.75	1.24	**0.680**
10. I should fix my own problems rather than telling them to other people	3.08	1.25	**0.628**
12. I never let others know how hard I work on things	3.17	1.18	**0.445**
**Non‐disclose**	12.48	3.31	**–**
	61.21	11.33	**–**

**FIGURE 1:**
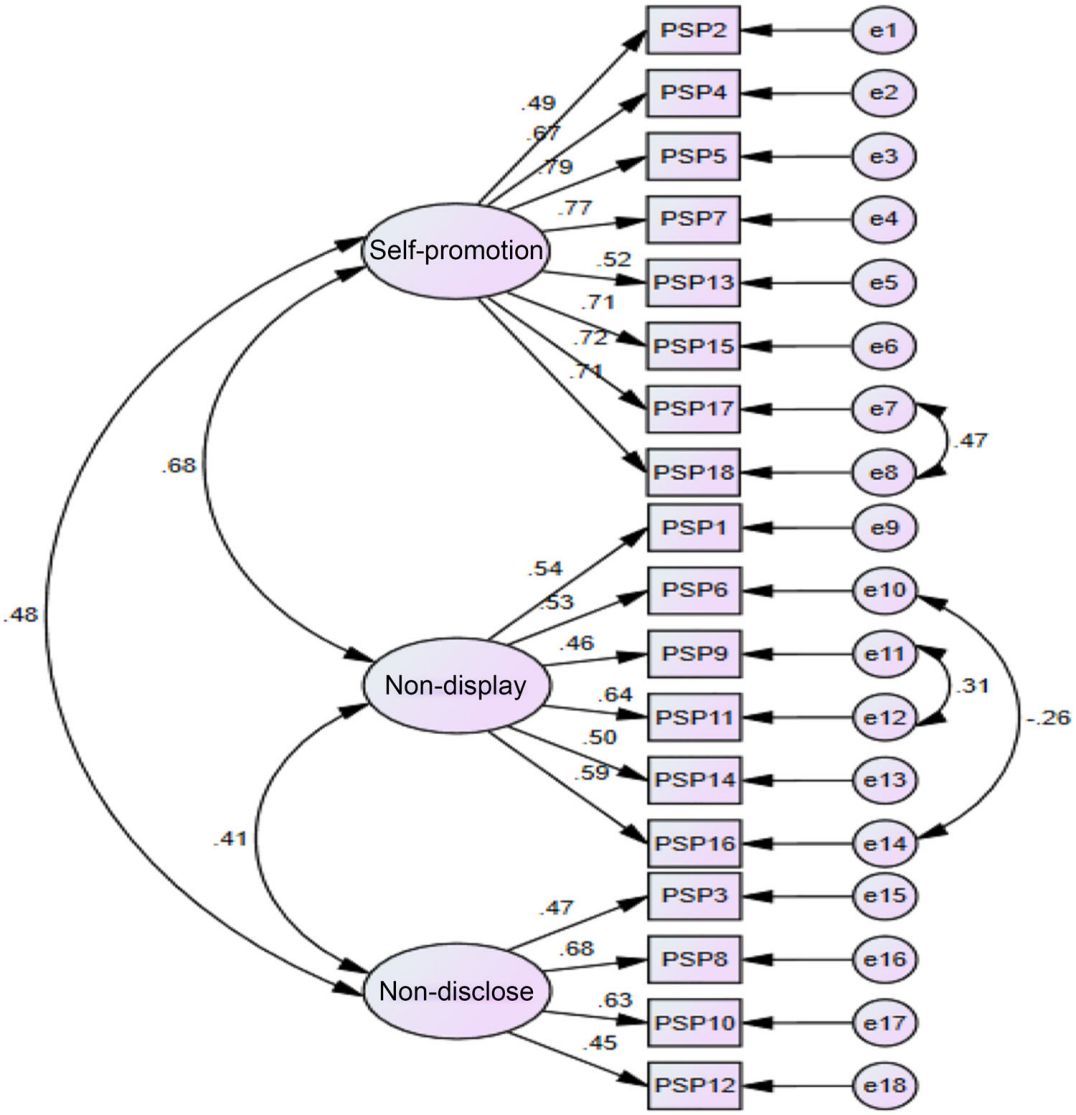
Confirmatory factor analysis of perfectionistic self‐presentation scale (PSPS).

Goodness‐of‐fit indices were used to measure the validity of the model. The comparative fit index (CFI), Tucker–Lewis index (TLI), and incremental fit index (IFI) are acceptable when above 0.90. Moreover, a model is fit when the values of the parsimony comparative fit index (PCFI) and parsimony normed fit index (PNFI) are higher than 0.50. Although *X* ^2^ is often the most reliable index to evaluate the goodness of fit, it is correlated with increased sample size and degree of freedom and may not be confirmed in most cases. Instead, researchers usually use the root mean square error of approximation (RMSEA) and CMIN/DF. Thus, a model is fit when RMSEA is lower than 0.08 and CMIN/DF is less than 3 (Kline, 2015). Meanwhile, the primary indices of the model did not represent good fitness, and covariance was added to the remaining values to enhance the fitness of the model. For this purpose, covariance was created between errors 7 and 8 related to the self‐promotion component and errors 10 and 14 and 11 and 12 related to the non‐display of imperfection component (see Figure 1).

Finally, the assessment of the fitness indices showed that the measured model fits properly (RMSEA = 0.062, CMIN/DF = 2.301, PNFI = 0.727, PCFI = 0.773, IFI = 0.917, TLI = 0.901, CFI = 0.916).

### 3.5 Convergent validity and reliability

To test CV, we used the average variance extracted (AVE) method. Moreover, composite reliability (CR) and Cronbach's alpha coefficient were used to evaluate the internal consistency reliability of four factors (i.e., perfectionistic self‐presentation, perfectionistic self‐promotion, non‐display of imperfection, and nondisclosure of imperfection). According to Fornell and Larcker (1981), higher CR values than 0.70 indicate the acceptable internal consistency of a construct. Further, higher AVE values than 0.50 confirm CV (Fornell & Larcker, 1981).

The overall analysis of the results showed that although the PSPS lacks acceptable CV, its CR is standard. Moreover, Cronbach's alpha coefficient of .866 confirmed the reliability of the PSPS (Table 2).

**TABLE 2:** The average variance extracted (AVE), composite reliability (CR), and Cronbach's alpha

Variable	AVE	CR	Cronbach's alpha
Self‐promotion	.466	.872	.873
Non‐display	.301	.718	.711
Non‐disclose	.318	.644	.635
PSP	.361	.744	.866

Abbreviation: PSP, perfectionistic self‐presentation.

### 3.6 Concurrent validity

To examine concurrent validity, we evaluated the correlation of the PSPS and its factors with each other, as well as with the CAPS and DAS‐CA. The obtained results showed that the PSPS, its factors, the CAPS, and the DAS‐CA were correlated positively and significantly; therefore, the concurrent validity of the scale is acceptable (Table 3). In general, it can be concluded that the PSPS has standard psychometrics.

**TABLE 3:** Correlations between research variables

	Self‐promotion	Non‐display	Non‐disclose	PSP	CAP	DA
Self‐promotion	1					
Non‐display	0.568^**^	1				
Non‐disclose	0.365^**^	0.298^**^	1			
PSP	0.896^**^	0.798^**^	0.616^**^	1		
CAP	0.566^**^	0.562^**^	0.307^**^	0.630^**^	1	
DA	0.420^**^	0.227^**^	0.324^**^	0.422^**^	0.391^**^	1

Abbreviations: CAP, child and adolescent perfectionism; DA, dysfunctional attitudes; PSP, perfectionistic self‐presentation.

**p < .01.

## 4 DISCUSSION

The present study aimed to measure the psychometrics of the PSPS–JR in an Iranian population. This is the first study to examine the statistical features of the Persian version of this scale, proposing results that can promote the intercultural applications of the PSPS–JR. In terms of face validity and content validity, our findings indicated that the PSPS–JR questions are suitable for the Iranian community, and none of the items need to be eliminated/corrected. Using construct validity, we assessed the data of the PSPS–JR using maximum likelihood confirmatory factor analysis. The CV of the scale was also measured and confirmed (AVE > 0.50). To evaluate the reliability and internal consistency of the scale, we used CR and Cronbach's alpha coefficient, and the results showed the acceptable internal consistency and CR of the scale. The analysis of the correlational matrix of the research variables indicated that the PSPS–JR is positively correlated with the CAPS and DAS‐CA.

These data accord with evidence from Hewitt et al. (2011) research. Hewitt et al. (2011) stated that rather than describing a trait dimension of perfectionism, this domain of perfectionism represents an individual's “interpersonal expression” of their perfection. The authors hypothesized that perfectionistic self‐presentation was made up of three facets, reflecting the structure of their original measure in adults. The authors developed 34 items, some derived from the PSPS for adults, and others informed by research in children and adolescents. The intercorrelations between the subscales were considered moderately high. Respectable‐to‐very good internal consistency was found across three different samples. To examine its validity, the authors tested the PSPS–JR against the CAPS, the results indicating that perfectionistic self‐presentation was correlated with, but distinct from, measures of SOP and SPP. The study also found perfectionistic self‐presentation to be associated with maladaptive outcomes in both clinical and nonclinical samples, accounting for a significant level of variance in both measures of depression and anxiety. Additionally, in line with Fellet et al. (2012) research, psychometric analyses indicated that two of the PSPS–JR subscales had acceptable levels of internal consistency, but the nondisclosure of imperfection subscale had relatively low internal consistency. The four‐item nondisclosure of imperfections subscale had unacceptable internal consistency. Perfectionistic self‐presentation had moderate‐to‐substantial associations with different measures of social anxiety and accounted for a significant amount of variance in the measure of dysfunctional attitudes, providing support for the importance of measuring perfectionistic self‐presentation in addition to trait‐based perfectionism to guide interventions when examining the maladaptive impacts of perfectionism.

The positive correlation of this instrument with CAPS and DAS‐CA provided evidence for its CV. Evidence for the concurrent validity of the PSPS–JR subscales was found in that these subscales were associated with perfectionism dimensions. In addition, there were robust associations established between all three PSPS–JR subscales and the measure of dysfunctional attitudes. These findings are consistent with the studies by Huang et al. (2020), Fellet et al. (2012), Hewitt et al. (2011), and Friedman (2006). Perfectionistic self‐presentation leads the individual to set high personal standards, which diminishes his/her self‐esteem; as a result, the individual may fear the consequences of success, and fear of success may trigger self‐defeating, avoidance behaviors (Wang et al., 2022). These behaviors amplify dysfunctional attitudes as such attitudes involve rigid, perfectionistic standards that the individual uses to judge him/herself, or others. As these attitudes are excessively rigid and resistant to change, they are considered dysfunctional (Huang et al., 2020). Thus, these attitudes may very well be linked to perfectionistic self‐presentation. As a personality trait, perfectionistic self‐presentation propels the individual to have high expectations, which, in turn, increases his/her dysfunctional attitudes and perfectionism.

Generally, PSPS–JR can be used to identify the essence and the consequences of perfectionistic self‐presentation in adolescents. Further, it allows psychologists and counselors to prevent the development of this personality trait. Overall, the PSPS–JR appears to be a useful measure of the expression of perfection among Iranian youths and an important tool in attempting to understand the nature and the consequences of perfectionistic self‐presentation in children and adolescents. The current findings have important practical implications. Inclusion of the PSPS–JR should enhance clinical assessments seeking to establish the nature of dysfunctional perfectionism in children and adolescents.

The results of the present study should be interpreted with caution due to some limitations we faced. First, the reliability of the research was measured based on Cronbach's alpha without a retest. Moreover, as the samples were selected from Tehran only, the results cannot be generalized to all Iranians. Therefore, a retest and further investigation on different samples are suggested to ensure the psychometrics of the PSPS in the Iranian population. Throughout the study, we adhered to ethical principles by guaranteeing privacy and secrecy (the questionnaires were completed anonymously). Moreover, participation was voluntary, and the participants were allowed to withdraw from the study at any given time.

## AUTHOR CONTRIBUTIONS

**Faezeh Peimanpak and Abbas Abdollahi**: Study design; data collection; data analyses; writing draft. **Simin Hosseinian**: Reviewed the manuscript; edited the manuscript

## CONFLICT OF INTEREST STATEMENT

The authors have no conflict of interests to disclose.

### PEER REVIEW

The peer review history for this article is available at https://publons.com/publon/10.1002/brb3.3074.

## CONSENT TO PARTICIPATE

Informed consent was obtained from all individual participants included in the study.

## Data Availability

The data are available by request to the corresponding author.
